# Metabolically Active, Fully Hydrolysable Polymersomes

**DOI:** 10.1002/anie.201814320

**Published:** 2019-02-27

**Authors:** Yunqing Zhu, Alessandro Poma, Loris Rizzello, Virginia M. Gouveia, Lorena Ruiz‐Perez, Giuseppe Battaglia, Charlotte K. Williams

**Affiliations:** ^1^ Chemistry Research Laboratory Department of Chemistry University of Oxford Oxford OX1 3TA UK; ^2^ Department of Chemistry and Institute of Physics of Living Systems University College London 20 Gordon Street London WC1H 0AJ UK; ^3^ EPSRC/Jeol Centre for Liquid Phase Electron Microscopy University College London London WC1H 0AJ UK; ^4^ Department of Chemical Sciences Faculty of Pharmacy University of Porto Portugal; ^5^ Institute for Bioengineering of Catalonia The Barcelona Institute of Science and Technology 08028 Barcelona Spain

**Keywords:** cells, drug discovery, polymers, ring opening, self-assembly

## Abstract

The synthesis and aqueous self‐assembly of a new class of amphiphilic aliphatic polyesters are presented. These AB block polyesters comprise polycaprolactone (hydrophobe) and an alternating polyester from succinic acid and an ether‐substituted epoxide (hydrophile). They self‐assemble into biodegradable polymersomes capable of entering cells. Their degradation products are bioactive, giving rise to differentiated cellular responses inducing stromal cell proliferation and macrophage apoptosis. Both effects emerge only when the copolymers enter cells as polymersomes and their magnitudes are size dependent.

Aliphatic polyesters can be biocompatible and biodegradable, and as such they are important materials for medical devices, tissue engineering, and in drug delivery.^1^ Three FDA approved and widely applied hydrophobic polyesters are poly(ϵ‐caprolactone) (PCL), polylactide (PLA), and poly(lactic‐*co*‐glycolic acid) (PLGA). By copolymerizing them with hydrophilic blocks it is possible to access amphiphiles that self‐assemble, in water, into micelles or vesicles (also known as polymersomes).1c, 2 In general, such supramolecular self‐assembly is a highly successful example of molecular bioengineering, providing control over particle size, architecture, surface chemistry, degradation rate, and mechanical properties.1c, 2a, 3

When designing nanocarriers there are many successful hydrophobic polymers to choose from, including aliphatic polyesters, carbonates, and peptides.^4^ In terms of hydrophilic blocks, poly(ethylene glycol) (PEG) is ubiquitous and forms nanostructures with prolonged blood circulation times, resulting in “stealth” delivery.^5^ For example, paclitaxel‐loaded PLA‐PEG micelles have been used in cancer treatment since 2007 and related PEG‐based nanoparticles are in late‐stage clinical trials.^6^ Nonetheless, PEG is not biodegradable and its use can cause renal accumulation and sensitivity.1b Polymeric alternatives to PEG are known but expanding the scope of new hydrophilic materials remains important.^7^ Here, a new polymer‐based nanomedicine concept is presented and it exploits fully degradable, amphiphilic block polyesters.

Amphiphilic polyesters have long been targeted but are very difficult to prepare by condensation polymerization methods. Such block polymers are best synthesized by controlled polymerizations and the well‐known method for polyesters, lactone ring‐opening polymerization (ROP), is most effective for hydrophobic blocks.^8^ It has been used to make a few hydrophilic polyesters but such processes require complex monomer syntheses, hydrophile protection/deprotection strategies, and may be hampered by low polymerizability.^9^ Recently, the ring‐opening copolymerization (ROCOP) of epoxides and anhydrides has emerged as a tolerant, functional‐group compatible synthesis, but so far applications for the resulting alternating polyesters are under‐explored.^10^ Here, new amphiphilic block polyesters are prepared by ϵ‐caprolactone (ϵ‐CL) ROP, followed by ROCOP of succinic anhydride (SA) and 2‐((2‐(2‐(2‐methoxyethoxy)ethoxy)ethoxy)methyl)oxirane (ME_3_MO; Figure 1 a; see Figure S2 in the Supporting Information). The polyesters are deliberately designed to degrade to metabolites.

**Figure 1:**
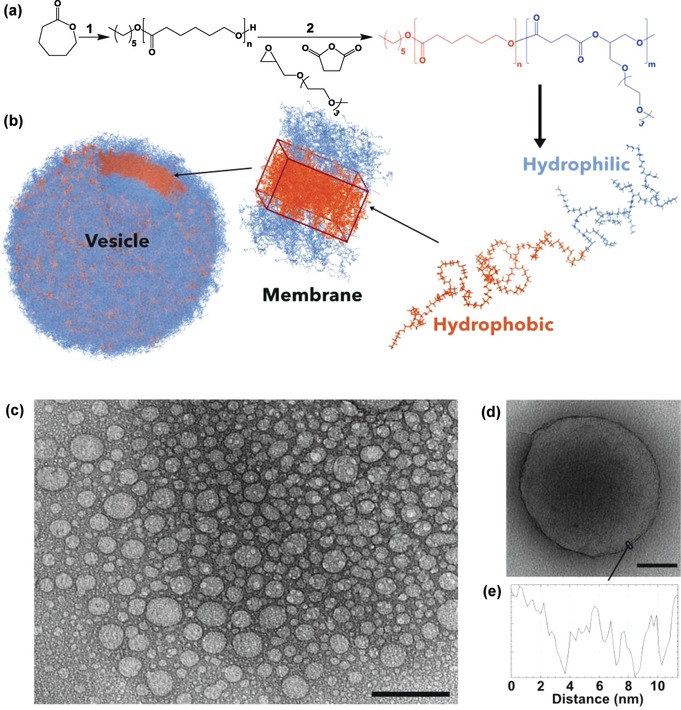
a) Synthesis of PCL‐*b*‐PE. For detailed reaction conditions, see Tables S1–S3. b) Copolymer chain minimized and assembled into a membrane which in turn encloses into a polymersome. c) TEM of a PCL_38_‐*b*‐PE_7_ polymersome dispersion: scale bar 500 nm. d) TEM of a single polymersome made of PCL_54_‐*b*‐PE_7_ and e) the corresponding details for the membrane (scale bar 100 nm).

First, two different PCL macroinitiators were prepared by ϵ‐CL ROP using an organocatalyst, and control of monomer/catalyst loadings afforded PCL_38_‐OH and PCL_54_‐OH (Table 1; see Table S1). The PCL samples showed narrow, monomodal molar mass distributions with masses, evaluated by both SEC and ^1^H NMR spectroscopy, in excellent agreement with theory.

**Table 1:** Polyester macroinitiators and amphiphilic block polyesters with variable compositions.

Polymer^[a]^	M_n,theo_^[b]^	M_n,NMR_^[c]^	M_n,SEC_ [Đ]^[d]^	*l*_c_ [nm]^[f]^	*D*_h_ [nm] (PDI)^[g]^	*t* [nm]^[h]^
PCL_38_‐OH	4.6	4.3	4.4 (1.15)^[e]^	–	–	–
PCL_54_‐OH	5.7	6.2	6.4 (1.20)^[e]^	–	–	–
PCL_38_‐b‐PE_7_	9.1	6.6	11.0 (1.13)	10.9	53±7 (0.38)	6.4±1.2
PCL_54_‐b‐PE_7_	11.0	8.4	16.5 (1.18)	14.4	398±13 (0.17)	8.6±1.4
PCL_54_‐b‐PE_5_	9.8	7.7	14.3 (1.20)	12.8	277±13 (0.24)	8.7±1.6

Note: The data are acquired using purified polymers (see the Supporting Information for details). [a] Polymerization conditions described in Tables S1–S3. PE is used to represent P(SA‐*alt*‐ME_3_MO). [b] Theoretical molar mass, Samples #1–2: M_n,theo_=([ϵ‐CL] × conversion × M_[ϵ‐CL]_)/[n‐hexanol]; Samples #3–5=([SA] × conversion × M_[SA+ME3MO]_)/([PCL‐OH] + [ϵ‐CL]). [c] Calculated from ^1^H NMR integrals (Table S3). [d] Determined by SEC, in THF, at 30 °C, calibrated using narrow MW polystyrene standards. [e] M_n_ values for PCL corrected with a coefficient (multiplied by 0.56).^11^ [f] Estimated size of a single polymer chain using the method PM7^12^ with an implicit solvent model COSMO^13^ and assuming dielectric constants of 78.4 and 4.0 for the hydrophilic and hydrophobic blocks, respectively. [g] Polydispersity index determined by DLS in deionized water with polymer ≈0.25 mg mL^−1^. [h] Hydrophobic membrane thickness measured by TEM.

For each sample, the degree of polymerization (PCL_*n*_) was calculated from the ^1^H NMR integrals for the polymer methylene signals against the chain end groups (see Figure S1). The PCL macroinitiators were subsequently used in the ROCOP of SA and ME_3_MO (Figure 1 a; see Figure S2).^14^ This reaction was catalyzed using a commercial Cr^III^ system [salenCr(Cl)/PPNCl] and was monitored, by aliquot analysis, and terminated when succinic acid conversion was greater than 80 %. One drawback is that the cocatalyst (PPNCl) delivers an alternative initiating group (Cl^−^) which contaminates the block polymer. To overcome the problem, a tenfold excess of macroinitiator was applied and residual alternating polymer was removed by repeated precipitations (see the Supporting Information for details). The purified block polymers, PCL‐*b*‐PE, all show higher *M_n_* values than the PCL precursors and narrow dispersities (see Figure S3). By controlling monomer loadings, it was straightforward to access amphiphiles with hydrophobic weight contents (PCL) from 66–79 wt % (Table 1). In all cases, block polyester formation was confirmed by ^1^H NMR spectra, SEC, ^31^P{^1^H} NMR end‐group titration, and DOSY NMR spectra (see Figures S3–S5).^15^

To rationalize the self‐assembly, calculations were conducted on the copolymer chains using a semi‐empirical method PM7^12^ with an implicit solvent model COSMO,^13^ assuming dielectric constants of 78.4 and 4.0 for the hydrophilic and hydrophobic blocks, respectively. These calculations indicate the size of a single chain, and consequently how it may pack in micelles or membranes (Table 1). Using the results of the simulations and applying the general theory of block polymer assembly,^15^ the PCL chain length was estimated as *l*_PCL_=0.63 *N*_PCL_^0.66^ [where 0.63 (nm)=average caprolactone monomer length]. Similarly, assuming a fully stretched conformation for the hydrophilic block,^16^ *l*_PE_=0.8*N*_PE_ [0.8 (nm)=average SA‐ME_3_MO monomer length].

PCL‐*b*‐PE self‐assembly was performed using a solvent‐switch method, whereby the copolymer is initially dissolved in a good solvent for both blocks and then gradually exchanged with water (see the Supporting Information for details). Formulation characterizations, in terms of size and polydispersity, were assessed using dynamic light scattering (DLS; see Figures S6 and S7).^17^ The hydrodynamic diameters (*D*_h_) varied depending on the polyester building block composition and overall molar mass (see Figure S6). The DLS measurements suggest the formation of spherical structures whose radius is considerably larger than a single chain length. This feature implies that the copolymers assemble into membranes that in turn form into spherical vesicles. Transmission electron microscopy (TEM) was used to confirm the vesicular structure (Figure 1 c). All the samples appeared spherical, with varied diameters, in agreement with DLS measurements, and moreover support vesicle formation. TEM measurements also enable estimation of the membrane thickness and values increase with PCL block length (Table 1).^15^ It is notable that the vesicle size depends on a range of variables and is also dependent upon the self‐assembly preparation method. Here, the polymersomes are prepared by the same method and by controlling polymer composition and molar mass, and two distinct polymersome populations are produced with considerable differences in size.

Biodegradability studies were conducted using *Pseudomonas cepacia* lipase.^18^ Experiments were monitored using DLS (see the Supporting Information for details). Over the first 30 minutes, a rapid decrease in the *D*_h_ from about 400 to 200 nm was accompanied by an increase in polydispersity (see Figure S9). As the mean count rate from DLS is proportional to both the nanoparticle number and size, provided the attenuator is fixed, its value was used to infer the rate of enzymatic hydrolysis.^19^ Over the first 30 minutes, the mean count rate decreased rapidly from 151 to about 50 kcps (attenuator value=6; Figure 2). Whereas a control experiment without lipase showed almost the same mean count rate value over the equivalent period (see Figure S9). These results suggest that the lipase catalyzes the polyester hydrolysis, causing polymersome disassembly and polymer dissolution. The polymer degradation monitored by SEC reveals that from 2–4 hours, the overall molar mass decreased from 16.5–1.4 kg mol^−1^ together with increased *Ð* (see Figure S10 and Table S4). After 4 hours, polymer signals are no longer detectable, indicating degradation (see Table S4). The degradation products were identified as succinic acid, 6‐hydroxyhexanoic acid, and the glycerol derivative with tri(ethylene glycol) substituents using ^1^H NMR spectroscopy (see Figure S11).

**Figure 2:**
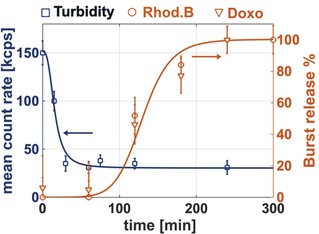
Mean count rate (blue squares) versus time for polyester polymersome solutions in the presence of lipase. Drug burst release profiles for RhB (orange circles) and DOX⋅HCl (orange triangles) loaded vesicles, representing the fits of the cumulative release profiles for the drug‐loaded polymersomes compared to the free drug release across the dialysis membrane.^21^ (see Figures S13 and S14 for cumulative drug release profiles).

The polymersome's drug loading/release profiles were evaluated using either rhodamine B (RhB) or doxorubicin hydrochloride (DOX⋅HCl) as water‐soluble model drugs (see Figures S12–S14).^20^ In vitro drug release studies were performed by dialysis against a phosphate buffer with or without lipase, at 37 °C and pH 7.4 (see the Supporting Information for details). The cumulative release profiles for RhB‐ or DOX⋅HCl‐loaded polymersomes exhibited broadly similar behaviors (see Figures S13 and S14). In the absence of lipase and after 20 hours, the cumulative drug release reached about 70 % compared to quantitative release from the free drug control. When lipase was added, drug release was almost complete within 1 hour (Figure 2). These release experiments are fully consistent with the observed polymer degradation rates.

As mentioned, the PCL‐*b*‐PE is biodegraded into three well‐defined compounds, each of which is known to be metabolizable.^22^ To understand the polymersomes’ and degradation products’ cytotoxicity, inflammatory, and immune responses various in vitro cellular studies were conducted. The materials were exposed to three cell lines, including cancerous human oral carcinoma (FaDu), acute leukemia monocyte‐derived macrophages (MΦ), and healthy primary dermal fibroblasts (HDF). As the cell‐line selection includes both professional (MΦ) and non‐professional (FaDu and HDF) phagocytes, the polymersomes’ cellular uptake profile was evaluated using confocal image analyses (Figure 3 a). They were all successfully internalized by all cells within 24 hours, with maximum uptake occurring over 48 hours (Figures 3 b; see Figure S15). Image quantification analyses showed that while HDF and FaDu cells share similar uptake profiles, the MΦ showed enhanced uptake after 48 hours. Cell viability was evaluated using a metabolic assay (see the Supporting Information for details). FaDu cells were unaffected by all materials regardless of either concentration or incubation time (see Figure S16). MΦ treated with polymersomes showed significantly decreased viability (ca. 50 % at 28 μg mL^−1^), but were unaffected by the degradation products even after 48 hours (see Figure S16). This decreased viability may be correlated with the enhanced macrophage uptake. Polymersomes should be internalized by both *endo*‐ or phagocytosis (the latter being a special trait of MΦ),^23^ resulting in higher uptake. It is also known that increased succinic acid concentration can reduce the mitochondrial membrane potential, which in turn boosts production of reactive oxygen species (ROS).^24^ Thus, the increased metabolic activity could be related to such ROS (see Figure S16). In contrast, polymersome treatment increased HDF mitochondrial activity (see Figure S16) and hence cell proliferation as confirmed using a total cell counting assay (see Figure S17).

**Figure 3:**
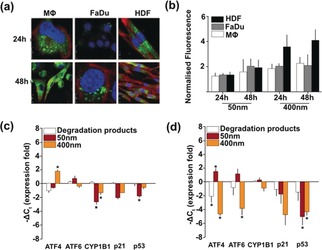
a) Confocal images of FaDu, MΦ, and HDF incubated with RhB‐polymersomes (398 nm, in green) for 24 and 48 h. The cell nuclei were stained with DAPI in blue and red CellMaskTM was used for cell membrane staining. b) Quantification of uptake of both polymersomes in HDF, FaDu, and MΦ over 48 h (*n*=3 independent experiments). c,d) The qPCR analyses for quantifying gene expression in HDF and MΦ, respectively. All experiments were carried out as three independent replicates, followed by *t*‐test statistical analyses (*p<0.05).

To understand the molecular bases for the differentiated cellular responses, gene expression profiles were evaluated by quantitative polymerase chain reactions (qPCR). The selected genes include cell cycle regulators p21 and p53, intracellular misfolded protein sensors ATF4 and ATF6, and general cell‐stress sensor CYP1B1. For HDF, after 24 hours of treatment with 398 nm polymersomes, an up‐regulation of an ATF4 sensor was induced, indicating possible formation of intracellular misfolded proteins (Figure 3 c). Conversely, in MΦ such an effect was observed for 53 nm polymersomes (Figure 3 d). Both qPCR analyses revealed that polymersomes induced a significant down‐regulation of p21 and p53, and could explain the improved HDF proliferation activity as these genes promote cell growth.^25^ Likewise, down‐regulation of CYP1B1 is a strong indicator of general cellular stress.

Moreover, MΦ treated with either 398 nm polymersomes or degradation products showed down‐expression of ATF4, ATF6, p21, and p53 (Figure 3 d). Thus, in line with the metabolic activity studies, suggesting induced cell apoptosis (see Figure S16). To understand whether increased cell stress could be related to an inflammation process, the nuclear translocation of the nuclear factor kappa B (Nf‐kB) was evaluated.^26^ Nf‐kB is an effector protein that transfers from the cytosol to the nucleus at the start of inflammation.^26^ Upon binding to conserved DNA regions the transcription of inflammation‐related genes, like cytokines and chemokines, occurs.^26^ No enhanced Nf‐kB nuclear translocation was observed for any of the materials even after 48 hours (see Figure S18). This observation indicates that the decreased MΦ viability is most likely a result of succinic acid promoted production of ROS, resulting from intrinsic cell phagocytosis. Taken together, these in vitro cellular results support two main hypotheses: the PCL‐*b*‐PE polymersomes induce the hyper‐proliferation of fibroblasts but reduce viability of macrophages, and both are important outcomes in most healing processes. Further, both effects correlate with supramolecular structure and size.

In summary, new amphiphilic and degradable block polyesters were prepared in high yield using controlled polymerizations, which allow easy control of composition. They degrade to metabolites including succinic acid, 6‐hydroxyhexanoic acid, and a derivative of glycerol. Degradable polymer nanostructures are important in current and future drug delivery, yet their bioactivity and that of degradation products remains rather poorly understood. This work demonstrates the potential to exploit polymers and degradation products to modulate cell behavior, for example, stimulating the proliferation of dermal fibroblasts. These nascent materials designed for metabolic activity should be optimized in future for selective drug delivery, cell‐specific wound healing, or targeted tissue engineering. This work highlights, for the first time, the scope for new alternating polyesters both as polymer hydrophiles and for future medical applications. Given the broad range of commercially available and functionalized epoxides/anhydrides and their high thermodynamic polymerizability, many other block polymers should be accessible using the methods demonstrated here. It is also straightforward to control the chain end group chemistry, alternating side‐chain substituents, degree of hydrophilicity, and crosslinking, and all provide future opportunities for fine‐tuning desired bioactivities.

## Conflict of interest

The authors declare no conflict of interest.

## Supporting information

As a service to our authors and readers, this journal provides supporting information supplied by the authors. Such materials are peer reviewed and may be re‐organized for online delivery, but are not copy‐edited or typeset. Technical support issues arising from supporting information (other than missing files) should be addressed to the authors.

Supplementary
