# Supplementary material for: Metabolically Active, Fully Hydrolysable Polymersomes
Source: Angew Chem Int Ed Engl. 2019 Feb 27;58(14):4581–6. doi: 10.1002/anie.201814320 (PMC6492077; doi:10.1002/anie.201814320)
Supplement: Supplementary file 1 — Supplementary [file ANIE-58-4581-s001.pdf]

Angewandte Chemie
Eine Zeitschrift der Gesellschaft Deutscher Chemiker

Supporting Information

Metabolically Active, Fully Hydrolysable Polymersomes

Yunqing Zhu, Alessandro Poma, Loris Rizzello, Virginia M. Gouveia, Lorena Ruiz-Perez, Giuseppe Battaglia, and Charlotte K. Williams**

anie_201814320_sm_miscellaneous_information.pdf

Content

General Procedures 3
Table S1. 4
Table S2. 5
Table S3. 5
Figure S1. 10
Figure S2. 11
Figure S3. 11
Figure S4. 12
Figure S5. 13
Figure S6. 13
Figure S7. 14
Figure S8. 15
Figure S9. 16
Figure S10. 17
Table S4. 17
Figure S11. 18
Figure S12. 18
Figure S13. 19
Figure S14. 20
Figure S15. 21
Figure S16. 22
Figure S17. 23
Figure S18. 23
Figure S19. 24
Figure S20. 24
References 25

General Procedures

Materials

All solvents and reagents were obtained from commercial sources (Aldrich and Fisher) and used as received unless stated otherwise. ε-Caprolactone (ε-CL) was dried over calcium hydride and stored under nitrogen. THF and toluene were distilled from sodium and stored under nitrogen. 2-Chloro-4,4,5,5-tetramethyl dioxaphospholane and 1,5,7-triazabicyclo[4.4.0]dec-5-ene (TBD) were used as received. Succinic anhydride (SA) was recrystallized from benzene, prior to sublimation, under vacuum, at 100 °C. Bis(triphenylphosphine)iminium chloride (PPNCl) was purified by recrystallization from diethyl ether and acetonitrile. Dialysis tubing (14 kg mol^{-1} molecular weight cut-off) was obtained from BioDesign Inc. of New York.

Characterization*: *SEC. The polyesters were dissolved in SEC grade THF and filtered through a 0.2 μm syringe filter prior to analysis. SEC, Shimadzu LC-20AD, was used to characterize the molecular weights and dispersities. The instrument was equipped with a refractive index (RI) detector and two Mixed Bed PSS SDV linear S columns. THF was used as the eluent, at a flow rate of 1.0 mL/min at 30 °C. Narrow molar mass polystyrene standards were used to calibrate the instrument.

NMR. All NMR Spectra were recorded using a Bruker AV 400 MHz spectrometer.

DLS. Dynamic light scattering (DLS) was used to determine the hydrodynamic diameter (D_h) and polydispersity of the nanostructures formed from PCL-*b*-PE, in aqueous solution, and was measured using the Zetasizer Nano series instrument (Malvern Instruments Zen1600). The scattering angle was fixed at 173°. Data processing was carried out using cumulant analysis of the experimental correlation function and the Stokes−Einstein equation was used to calculate the hydrodynamic radii. All solutions were analyzed using disposable polystyrene cuvettes.

TEM. TEM imaging was performed using a JEOL JEM-2200FS microscope, equipped with a field emission gun (FEG) at 200 kV, and an in-column energy Omega filter. The microscope was used in energy-filtered transmission electron microscopy (EFTEM) mode in order to increase image contrast by collecting only elastically scattered electrons. The software used for image acquisition and processing was the Digital Micrograph™ software (version 3.20). Images were recorded using a direct detection camera K2 IS from Gatan working in linear mode. This camera allowed for beam sensitive materials to be imaged at low electron doses. 400 mesh copper grids were glow-discharged for 40 s to render them hydrophilic. Then, 5 μL of polymersome dispersions (concentration ~0.5 mg/mL) were deposited onto the grid for 1 min. The grid was blotted with filter paper and immersed in Uranyless (Delta Microscopies, France) staining solution for 40 s for negative staining. The grid was blotted again and dried under vacuum for 1 min.

Zeta Potential. Zeta potential studies were conducted from 20 °C to 85 °C using a ZETASIZER Nano series instrument (Malvern Instruments).

Fluorescence Spectroscopy. Fluorescence experiments were carried out to evaluate the drug-loading efficiency *via* a Cary eclipse Fluorescence Spectrometer (VARIAN). The drug release profiles were also monitored using fluorescence spectroscopy.

Synthesis of PCL-OH Macroinitiator

The procedure used for the two macro-initiators is illustrated for the synthesis of PCL_{38}-OH. The organo-catalyst TBD (10.0 mg, 7.20×10^{-2} mmol), ε-CL (5.0 mL, 43.1 mmol) and 1-hexanol (147.0 μL, 1.44 mmol) were dissolved in dry toluene (8.6 mL), under N_2 protection, in a Schlenk tube charged with a stir bar. The reaction mixture was allowed to react at 25 °C for 6.5 h. Then benzoic acid (200.0 mg, 1.64 mmol) was added into the mixture, under a N_2 flow to terminate the polymerization. The relative molar ratios of [TBD]/[1-hexanol]/[ε-CL] were 1/20/598. The crude PCL was purified by precipitation from the reaction solution by addition of MeOH (50 mL × 3) to yield the product as a white powder.

Table S1. Relative quantities of reagents used in Table 1, entry 1-2.

#	Molar ratio	Loading			t (h)
	[TBD]/[n-hexanol]/[ε-CL]	TBD	n-hexanol	ε-CL	
1	1/20/598	10.0 mg	145.0 μL	5.0 mL	6.5
2	1/20/1042	10.0 mg	145.0 μL	8.3 mL	10.0

Polymerization conditions: $[\varepsilon\text{-CL}]_0$ = 5.0 M, [*n*-hexanol] = 0.17-0.10 M, [catalyst] = 8.5-5.0 mM, toluene, 25 °C, 6.5-10.0 h.

Typical Procedure for the synthesis of PCL-*b*-PE

[SalenCrCl] (10.0 mg, 1.58×10^{-2} mmol), PPNCl (9.1 mg, 1.58×10^{-2} mmol), PCL_{38}-OH macroinitiator (640.0 mg, 1.58×10^{-1} mmol), succinic anhydride (237.0 mg, 2.37 mmol) and ME_3MO (600.0 μL, 2.69 mmol) were dissolved in toluene (1.5 mL), in a Schlenk tube, charged with a stir bar inside the glovebox. The reaction mixture was allowed to react at 80 °C for 6 h. Aliquots were taken regularly to monitor the conversion of succinic anhydride (~80 %). Polymerization was stopped before complete conversion of succinic anhydride. The relative molar ratio of [salenCrCl]/[PPNCl]/[PCL-OH]/[succinic anhydride]/[ME_3MO] were 1/1/10/200/220. The crude polymer was purified by precipitation repeatedly from MeOH (50 mL × 5), to remove any (co)catalyst and residual monomers. Since free PE is soluble in MeOH, precipitation from MeOH can also effectively remove any residual material. After precipitation from MeOH for 5 times, the ratio of the integrals for PCL and PE remained constant in the ^{1}H NMR spectrum, indicating successful removal of residual PE.

Table S2. Relative quantities of reagents used in Table 1, entry 3-5.

#	Molar ratio	Loading					t (h)
	[SalenCrCl]/[PPNCl]/ [PCL-OH]/[SA]/[ME_3MO]	SalenCrCl	PPNCl	PCL-OH	SA	ME_3MO	
1	1/1/10/200/220 (PCL_{38}-OH)	10.0 mg	9.1 mg	640.0 mg	237.0 mg	600.0 μL	9.0
2	1/1/10/200/220 (PCL_{54}-OH)	10.0 mg	9.1 mg	910.0 mg	237.0 mg	600.0 μL	9.0
3	1/1/10/150/170 (PCL_{54}-OH)	10.0 mg	9.1 mg	910.0 mg	178.0 mg	465.0 μL	6.0

Polymerization conditions: $[SA]_0$ = 1.0 M, [SalenCrCl]/[PPNCl]/[PCL-OH]/[SA]/[ME_3MO] = 1/1/10/(150-200)/(170-220), toluene, 80 °C, 6.0-9.0 h.

Table S3. Detailed polymer data corresponding to Table 1.

#	Polymer	wt% PCL	$M_{n,theo}$ (kg·mol^{-1})^{a}	$M_{n,NMR}$ (kg·mol^{-1})^{b}	$M_{n,SEC}$ (kg·mol^{-1})^{c}	$Đ$
1	PCL_{38}-OH	100	4.6	4.3	4.4^{d}	1.15
2	PCL_{54}-OH	100	5.7	6.2	6.4^{d}	1.20
3	PCL_{38}-*b*-PE_7	66	9.1	6.6	11.0	1.13
4	PCL_{54}-*b*-PE_7	73	11.0	8.4	16.5	1.18
5	PCL_{54}-*b*-PE_5	79	9.8	7.7	14.3	1.20

a) Theoretical molar mass, Samples #1-2: $M_{n,theo}$ = ([ε-CL] × conversion × $M_{[ε\text{-}CL]}$)/[n-hexanol]; Samples #3-5 = ([SA] × conversion × $M_{[SA+ME3MO]}$)/([PCL-OH] + [CL]); b) Calculated on the basis of the ^{1}H NMR integrals, the methyl end group (0.95 ppm) was used as the internal reference for #1-2 [comparing the integrals of peaks a (0.95 ppm) and c (2.36 ppm) in Figure S1A], while the methylene signal of the macroinitiator, PCL-OH, was used as internal reference for #3-5 [comparing the integrals of peak c (2.24 ppm) and peak h (2.57 ppm) in Figure S1B]; c) Determined by SEC, in THF, at 30 °C, calibrated using narrow molar mass polystyrene standards; d) M_n values are corrected (multiplied by 0.56).

Procedure for calibrating the quantity of DOX·HCl in PBS solution

The procedure was previously reported using tris buffer.[1] In this work, a stock PBS solution (10.0 mM, pH 7.4) of DOX·HCl (500 μg·mL^{-1}) was carefully diluted to a range of concentrations (0.5, 1.0, 2.0, 3.0 and 4.0 μg·mL^{-1}) using PBS solution (10.0 mM, pH 7.4). The fluorescence intensities of these samples were measured using the Fluorescence spectrometer. The PMT (*photomultiplier* tube) voltage was set to be 570 V and the excitation and emission slits were both 5 nm. Emission scans from 500~800 nm were collected, with λ_{ex} fixed at 461 nm. The peak intensities at 591 nm were used to prepare the calibration. Calibration data was linearly fit in Origin 8.1 and is reported in Figure S14B. The same instrument settings were used for the investigation of drug release profiles.

Colloidal self-assembly, drug-loading and in vitro release procedures.

The loading of model drugs (either rhodamine B (RhB) or doxorubicin hydrochloride (DOX•HCl)) were performed according to the following protocol. The polymer stock solution (2.00 mL, 1.25 mg mL^{-1} in THF) was mixed with a drug stock solution (0.50 mL, 0.05 mg mL^{-1}). Then, DI water (5.00 mL) was added, dropwise, to form the polyester polymersomes. The organic solvent was removed by flushing the solution with N_2 for 2 h. The residual THF and free drug (not encapsulated) were removed *via* dialysis using a dialysis tube (MWCO = 14 kg·mol^{-1}) against PBS solutions (1.0 L) at 25 °C. The PBS solution was renewed 6 times every 30 min and the concentration of the PBS solutions was increased gradually from 1.0 mM, 2.0 mM, 4.0 mM, 6.0 mM, to 8.0 mM and finally 10.0 mM, to prevent any sudden change in osmotic pressure across the polymersome membrane. Thereafter, the obtained solution was weighed and the fluorescence characterization was conducted to determine the drug loading efficiency (DLE) according to the following equation.

$$\text{DLE (\%)} = \frac{M_\text{e}}{M_\text{f}} \times 100\% \quad (1)$$

Where M_e represents the weight of drug encapsulated in polymersomes, M_f stands for the starting weight of drug added to the solution.

The *in vitro* drug release profile was measured in the following way. The DOX or RhB loaded polymersome solution (5.00 mL) was transferred immediately, to a clean dialysis tube (MWCO = 14 kg·mol^{-1}) and dialyzed against PBS solution (100.00 mL of a 10.00 mM solution at pH 7.4) at 37 °C under constant stirring in the dark. Additionally, drug loaded polymersomes were also dialysed against PBS solution (100.00 mL of a 10.00 mM solution at pH 7.4) in the presence of *Pseudomonas cepacia* lipase (concentration inside the dialysis tube, 100 unit mL^{-1}), at 37 °C, under constant stirring and in the dark. In the control experiment, a PBS solution containing drug was prepared first. The drug concentration was adjusted to be similar to that for the drug loaded polymersome solution, based on the DLE measured by the fluorescence spectroscopy. The drug solution (5.00 mL) was transferred to a clean dialysis tube (MWCO = 14 kg·mol^{-1}) and dialyzed against PBS solution (100.00 mL of a 10.00 mM solution at pH 7.4) at 37 °C, under constant stirring. Aliquots were taken for fluorescence characterization (λ_{ex} = 461 nm for DOX and λ_{ex} = 510 nm for RhB) at pre-determined time intervals. The calibration for DOX and RhB emission (Figure S13a and S14a) was pre-determined and was applied to determine the cumulative release profiles of either DOX (Fig. S13c) or RhB (Fig. S14c) overtime. The burst release was determined by fitting the cumulative release profile for the drug-loaded polymersomes against the free drug release across the dialysis membrane.

All preparations were performed in sterile conditions and in biological safety cabinets (KS15-Thermo Fisher Scientific). The polymer stock solution (2.00 mL, 1.0 mg mL^{-1} in THF) was exposed to three 30 min cycles of UV light to remove any background bacterial contamination. Then, DI water (4.00 mL) was added via a syringe pump with vigorous stirring over a 40 min period, to form the polyester polymersomes. The organic solvent was removed by flushing the solution with N_2 for 2 h. The residual THF was removed *via* dialysis using a dialysis tube (MWCO = 14 kg·mol^{-1}) against a sterile PBS solution (2.0 L) at 25 °C, over 48 h (3 changes/day). The resulting solution was weighed and used for the cell uptake and toxicity studies.

Cell uptake, viability, imaging and inflammation investigations

Primary human dermal fibroblasts (HDF), oral carcinoma (FaDu), and human THP-1 monocytes cells were purchased from ATCC®. HDF and FaDu cells were cultured and maintained using Dulbecco's Modified Eagle Medium (DMEM) (Sigma-Aldrich®) containing: 10 (v/v) fetal calf serum, 2 mM L-glutamine, 100 mg ml^{-1} streptomycin and 100 IU ml^{-1} penicillin (Sigma-Aldrich®). Cells were cultured at 37 °C/95% air/5% CO_2. Cells were periodically sub-cultured using Trypsin-EDTA solution 0.25% (Sigma-Aldrich®) for the detachment process and centrifuged at 2000 rpm for 5 min for the pellet collection. THP-1 monocytes were cultured and maintained in RPMI-1640 medium (Sigma), supplemented with 10 mM HEPES buffer, and 10 (v/v) fetal calf serum, 2 mM L-glutamine, 100 mg ml^{-1} streptomycin and 100 IU/ml penicillin. THP-1 monocytes were differentiated to macrophages through incubation with 5 ng mL^{-1} of phorbol 12-myristate 13-acetate (PMA) for 48 hours.[2] We chose this PMA concentration as it has been found to not undesirable regulate genes expression.[3] For cell viability, the Thiazolyl Blue Tetrazolium Blue (MTT, Sigma) method was used. Briefly, cells were seeded at a concentration of 5 x 10^3 cells per well in a 96 well plate overnight (O.N.). Increasing concentrations of polymersomes (398 nm) were then added in the growth media, namely 5.6, 14 and 28 μg mL^{-1}, for 24 and 48 hours. The medium growth was then removed and an acidified solution of isopropanol was added to dissolve the water-insoluble MTT formazan. The solubilized blue crystals were measured calorimetrically at 570 nm (plate reader ELx800, BioTek). The cytotoxicity profiles of both polymersomes (398 nm) and degradation products were determined using the same method. The degradation products were obtained by: first, enzymatic hydrolysis of polymersome solutions, then removal of *Pseudomonas cepacia* lipase using centrifuge filtration (MWCO = 14 kg·mol^{-1}, which is much lower than the molar mass of *Pseudomonas cepacia* lipase (~ 34 kg·mol^{-1}) to ensure complete removal[4]). Assuming all mass was recovered from centrifuge filtration, the concentration of original polymersomes solutions was used as the concentration of degradation products. For cells total counting, HDF were seeded at a concentration of 1 x 10^4 cells per well in a 6 well plate overnight (O.N.) and then incubated with 28 μg mL^{-1} of degradation products, polymersomes (53 nm) and polymersomes (398 nm) for 24 and 48 hours. Cells were then detached

with trypsin/EDTA solution (5 minutes at 37 ºC), incubated with trypan blue for staining dead cells, and counted with an automated cells counter (BioRad).

For uptake quantification, THP-1 cells were seeded on glass-bottom petri dishes (ibidi) at a concentration of 5 x 10^3 cells per well, and incubated with rhodamine-labeled polymersomes (0.1 mg mL^{-1}) for 24 and 48 h, followed by 3 steps of PBS washing. Cells were then stained with DAPI (Sigma, for nuclear staining), and far-red CellMask (Life Technologies, for cell membrane staining) and imaged with confocal microscope (Leica SP8). For the uptake quantification, 10 different regions of the petri dishes were captured, and the polymersomes fluorescent signal has been normalized to the nuclear intensity signal (using ImageJ).

For the quantification of macrophages inflammation levels, the THP-1 blue NF-kB reporter cells (InvivoGen) were used. These cells are stably transfected and express a secreted embryonic alkaline phosphatase (SEAP) reporter gene driven by an IFN-beta minimal promoter fused to five copies of the NF- kB transcription factor, which promotes cytokines production. The translocation of NF-kB from the cytosol to the nucleus induces the release of the SEAP phosphatase, whose activity can be monitored calorimetrically. In our experiments, THP-1 blue NF-kB reporter cells were differentiated into macrophages as previously described, and then incubated with 200 ng mL^{-1} of bacterial lipopolysaccharide (LPS, Sigma) as positive control, and with 28 μg mL^{-1} of polymersomes (53 nm), polymersomes (398 nm), and degradation products, for 24 and 48 h. 20 μL of media were then removed and incubated with the incubation media, according to the manufacturer production. The quantification of media color change was quantified with a spectrophotometer (plate reader ELx800, BioTek) by reading at 570 nm.

Reverse transcription polymerase chain reaction (RT-PCR), and PCR assays

First, the polymersomes (53 nm), polymersomes (398 nm) and degradation products solutions were diluted to 28 μg mL^{-1} which is the maximum concentration used in the MTT investigation. Cultured cells, incubated for 24 h with polymersomes (28 μg mL^{-1}), were lysed and total RNA was collected by using RNeasy Mini Kit (Qiagen). RNA concentration was measured with NanoDrop spectrophotometer (Thermo). Complementary DNA (cDNA) was synthesized from every 1 µg of total mRNA in 20 µL volume per tube with QuantiTect Rev. Transcription Kit (Qiagen). The samples were then run in a standard agarose gel (1%) for RNA quality control check. For the PCR analyses, GAPDH and ACTB were used as reference genes.

Quantitative analysis was assessed with QuantiTect SYBR Green RT-qPCR Kit (Qiagen). The amplification process was done in 20 µL/tube, using the following steps: 95 °C for 5 min to make active the DNA polymerase, followed by 40 cycles of 95 °C (10 s) for denaturation, and 60 °C (30 s)

for combined annealing and extension for all primers. Melting curve was also acquired, to analyze the sample quality, from 55 °C to 99 °C, by increasing of 1 °C min^{-1}. Data were analysed *via* ΔΔCt value. -ΔΔCt was calculated as follows: ΔCt = CtKi67 - Ct GAPDH; ΔΔCt = ΔCt(treated) - ΔCt(control). The genes expression was analyzed using the following list of primers:

**Gene name**	**Primer sequences**
p21	For: GTGAGCGATGGAACTTCGAC<br>Rev: CAGGTCCACATGGTCTTCCT
p53	For: TGGCCATCTACAAGCAGTCA<br>Rev: GGTACAGTCAGAGCCAACCT
CYP1B1	For: CTCGAGTGCAGGCAGAATTG<br>Rev: TCCTTGGGAATGTGGTAGCC
ATF4	For: CCGCAACATGACCGAAATGA<br>Rev: CTTGCTGTTGTTGGAGGGAC
ATF6	For: CTGTTACCAGCTACCACCCA<br>Rev: GGAGCCAAAGAAGGTGTTGG

Enzymatic Biodegradation of the polyester polymersomes

The polyester polymersome solution (1.0 mL of a solution of concentration 0.42 mg mL^{-1}) was mixed with of *Pseudomonas cepacia* lipase solution (1.0 mL, 200 unit mL^{-1}). Control samples were also prepared by mixing the polyester polymersome solution (1.0 mL, 0.42 mg mL^{-1}) with DI water (1.0 mL). The mixtures were then placed on a shaker (Eppendorf ThermoMixer, 600 rpm at 37 °C) and aliquots were taken for DLS and SEC measurements at predetermined time intervals.

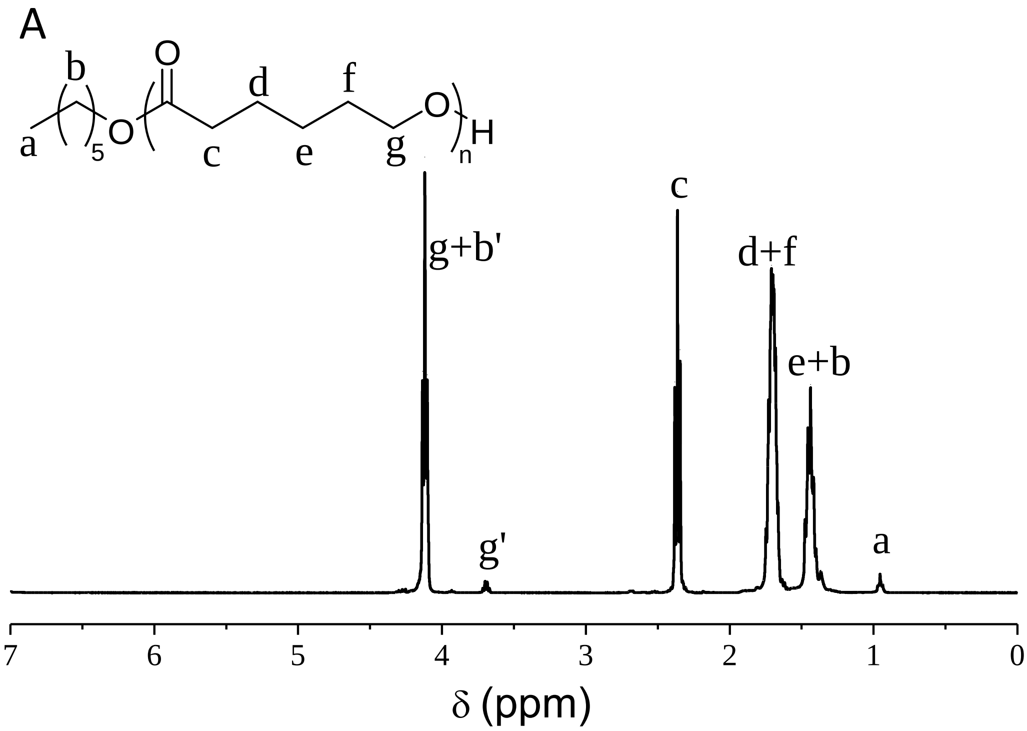

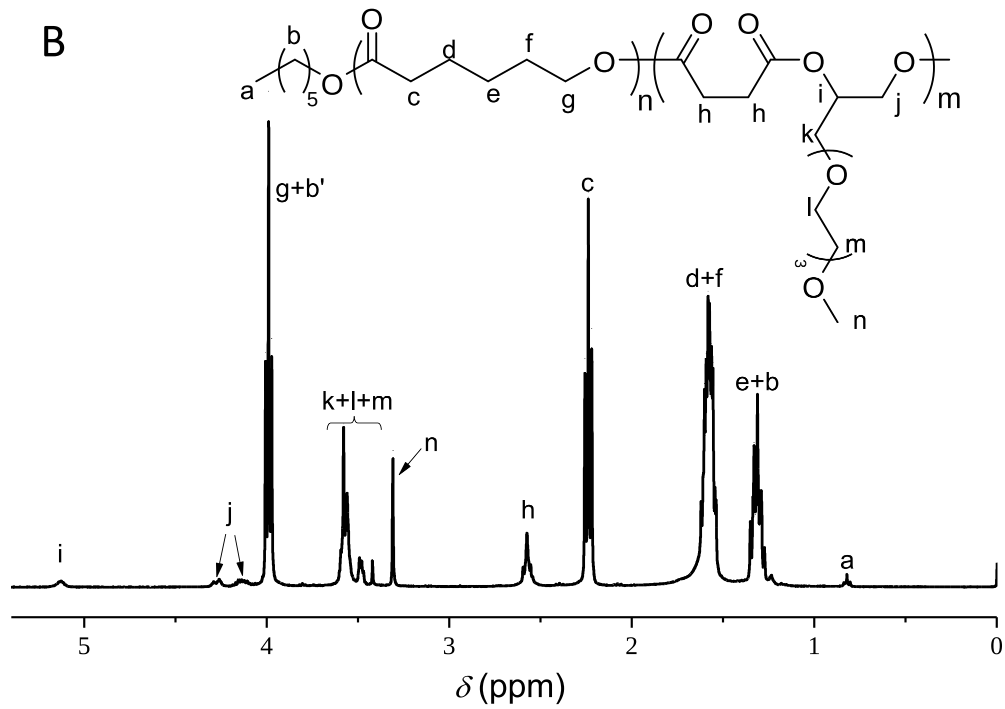

Figure S1. ^{1}H NMR spectrum of (A) PCL-OH macroinitiator (Table 1, #1) and (B) PCL-*b*-PE (Table 1, #3), in $CDCl_3$.

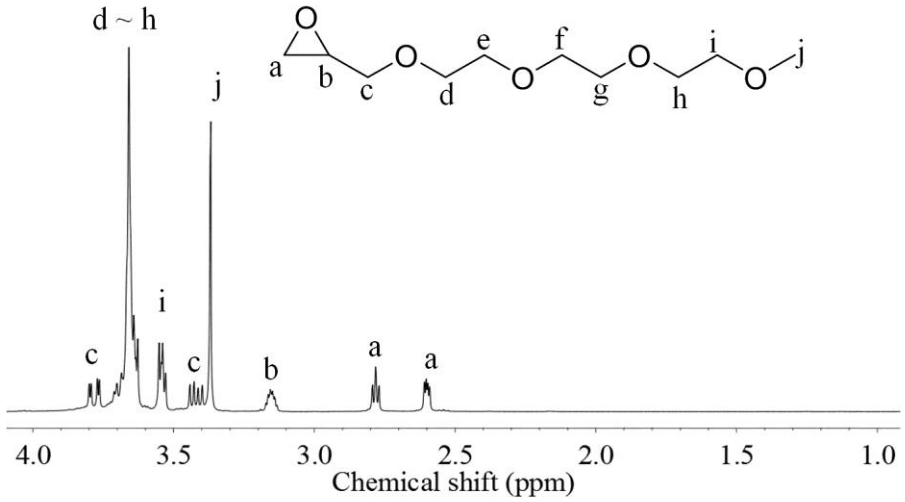

Figure S2. ^{1}H NMR spectrum of the epoxide monomer, ME_3MO, in $CDCl_3$.

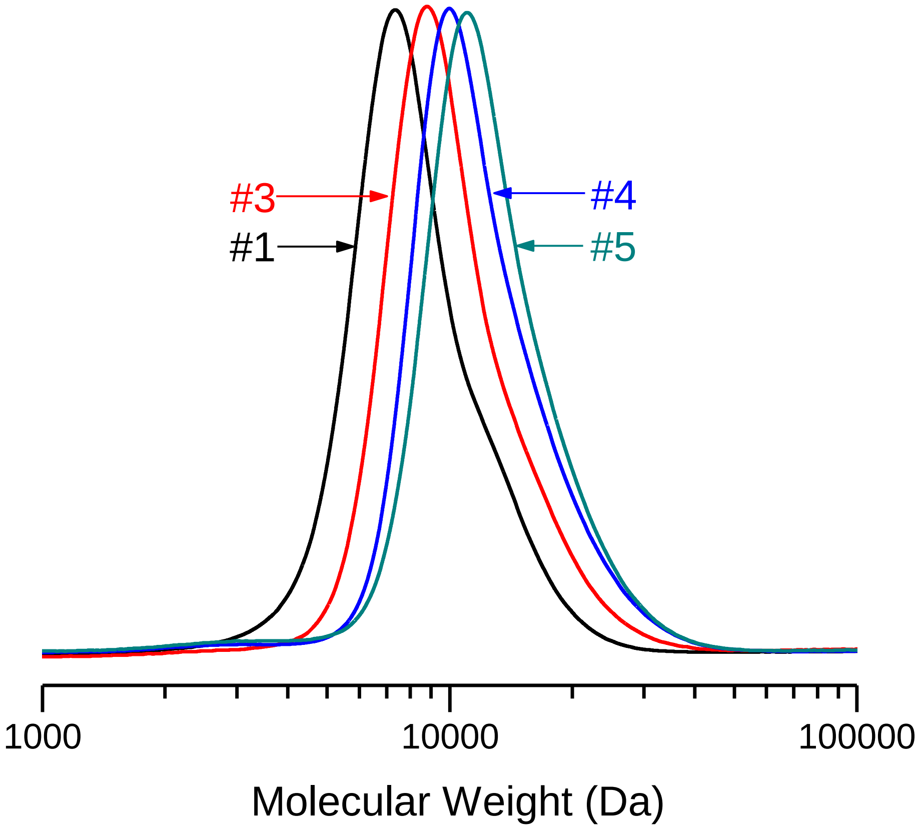

Figure S3. SEC traces of PCL-OH (Table 1, #1) and PCL-*b*-PE (Table 1, #3-5). The MWs are calibrated using narrow *Đ* polystyrene standards.

End group determination to confirm block structure

$^{31}P\{^{1}H\}$ NMR was used to monitor the polymer end groups for both PCL-OH and PCL-*b*-PE. The polymers were reacted excess 2-chloro-4,4,5,5-tetramethyl dioxaphospholane and bisphenol A was used as an internal reference.[5] PCL-OH shows a peak at 147.85 ppm, whilst PCL-*b*-PE shows two peaks at 147.05 ppm and 135.50 ppm, assigned to –OH and –COOH end groups (Figure S4).

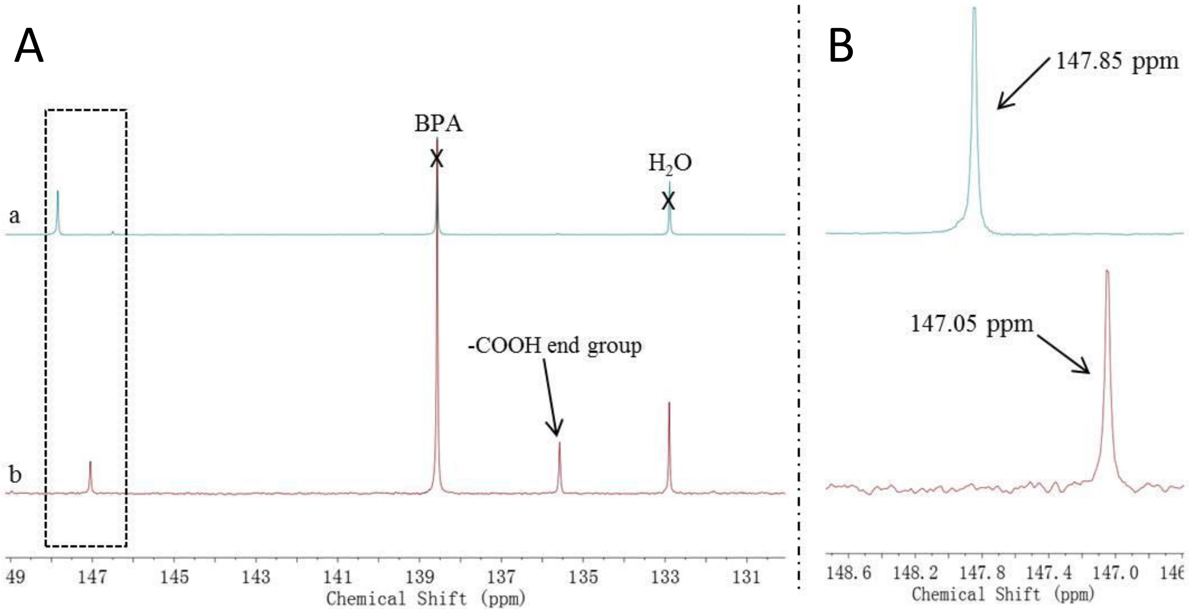

Figure S4. (A) $^{31}P\{^{1}H\}$ NMR spectra ($CDCl_3$) of (a) PCL-OH (Table 1, #1) and (b) PCL-*b*-PE (Table 1, #5) after being reacted with 2-chloro-4,4,5,5-tetramethyl dioxaphospholane (using bisphenol A as internal reference); (B) Enlarged $^{31}P\{^{1}H\}$ NMR spectrum corresponding to the highlighted area in (A). The signals at 147.85 ppm and 147.05 ppm are assigned to the primary –OH end groups of the PCL-OH macroinitiator and the –OH end group of PCL-*b*-PE block copolyester, respectively.

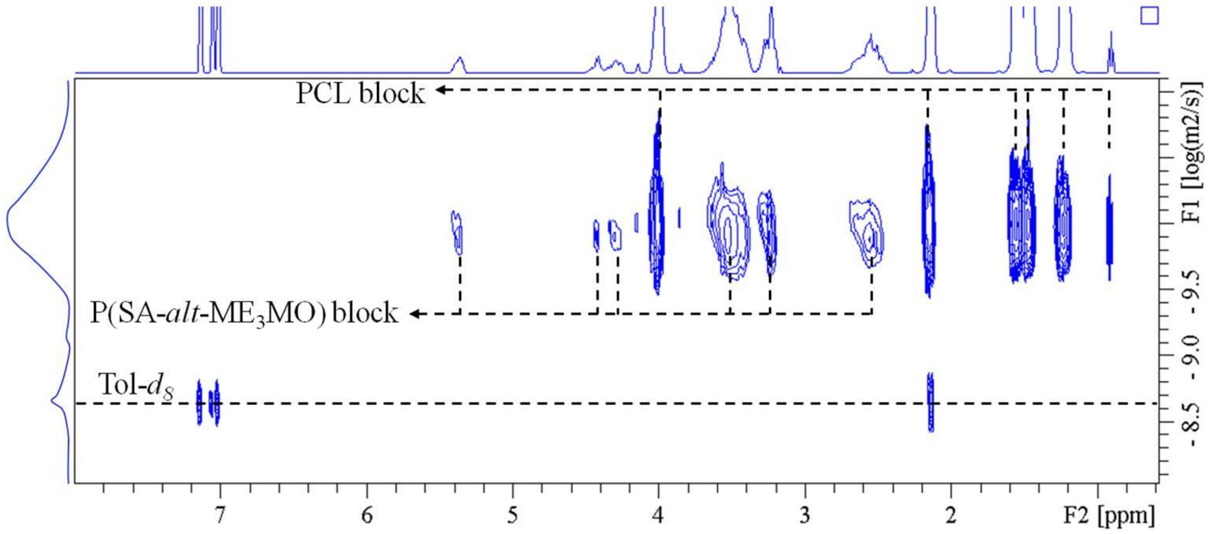

Figure S5. ^{1}H DOSY NMR spectrum of PCL-*b*-PE in toluene-d_8. (Table 1, #5).

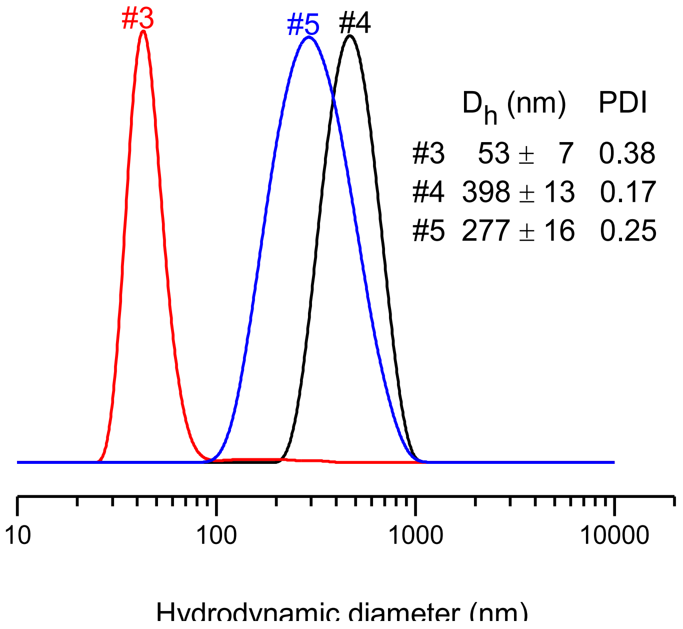

Figure S6. Dynamic light scattering analyses: hydrodynamic diameter (D_h) and polydispersity index (PDI) of the polymersomes formed from PCL$_{38}$-*b*-PE$_7$ (Table 1, #3), PCL$_{54}$-*b*-PE$_7$ (Table 1, #4) and PCL$_{54}$-*b*-PE$_5$ (Table 1, #5)

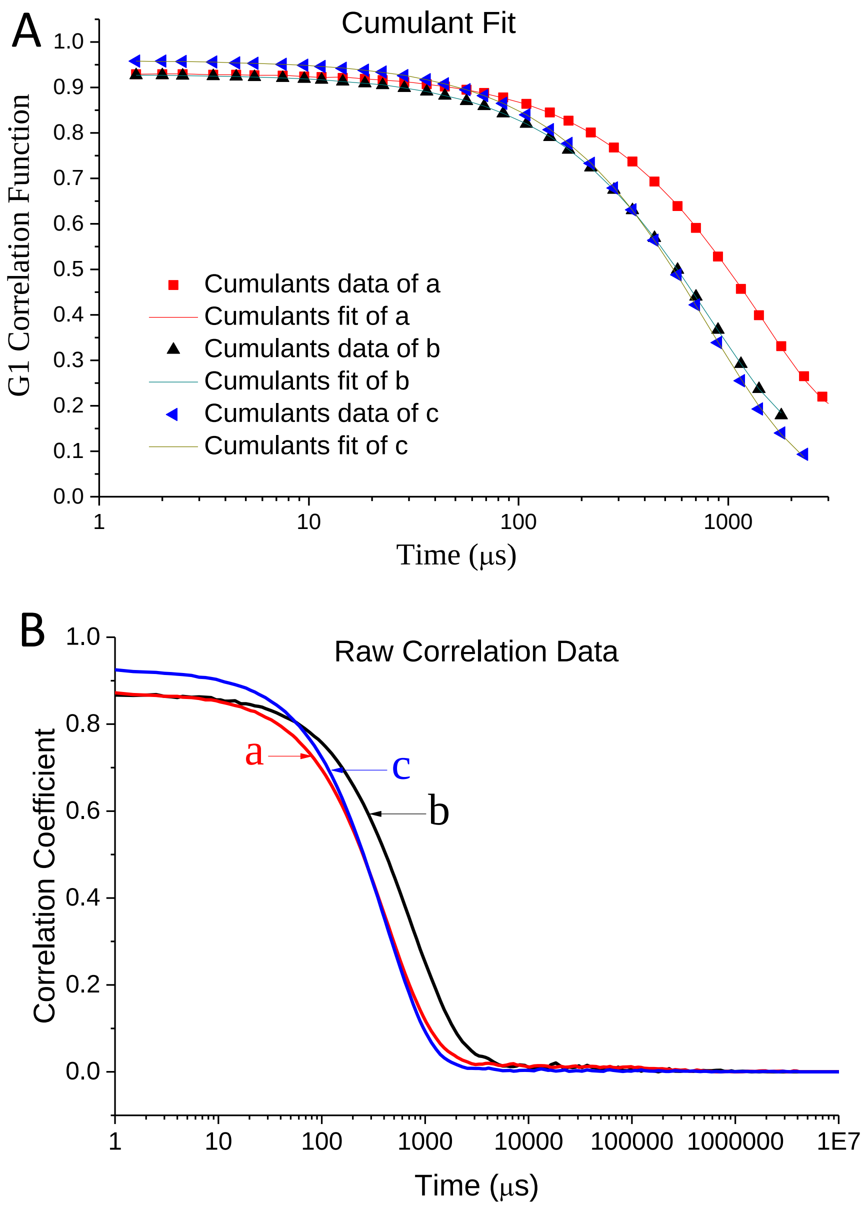

Figure S7. (A) Cumulant fit and (B) Correlation functions from the DLS experiments using the polymersomes (a, b and c correspond to the nanostructures formed from #3-5 in Table 1, respectively).

Zeta Potential Determination

The block polymer oligo(ethylene glycol) side chains are known to undergo a hydrophilic to hydrophobic transition when the polymer is heated above a particular temperature.[6] This loss of the hydrophilic corona is expected to significantly decrease the colloidal stability of the vesicles. Since the absolute value of the zeta potential is a measure of electrostatic repulsion between neighbouring colloidal nanoparticles and indicates colloidal stability,[22,27,34] the zeta potential of self-assembled nanostructures was monitored from 25 to 90 °C. Usually, colloidal systems having zeta potentials ranging from ±40 mV to ±60 mV are considered as showing 'good stability'.[7] Figure S8 shows both polymersomes (53 and 398 nm) show values ≤ –42 mV, at temperatures below 80 °C, indicating good colloidal stability at physiological temperature. The negative zeta potentials are attributed to the carboxylic acid end-groups on the hydrophilic block, which were also identified in the $^{31}P\{^{1}H\}$ NMR end-group titrations (Figure S4).

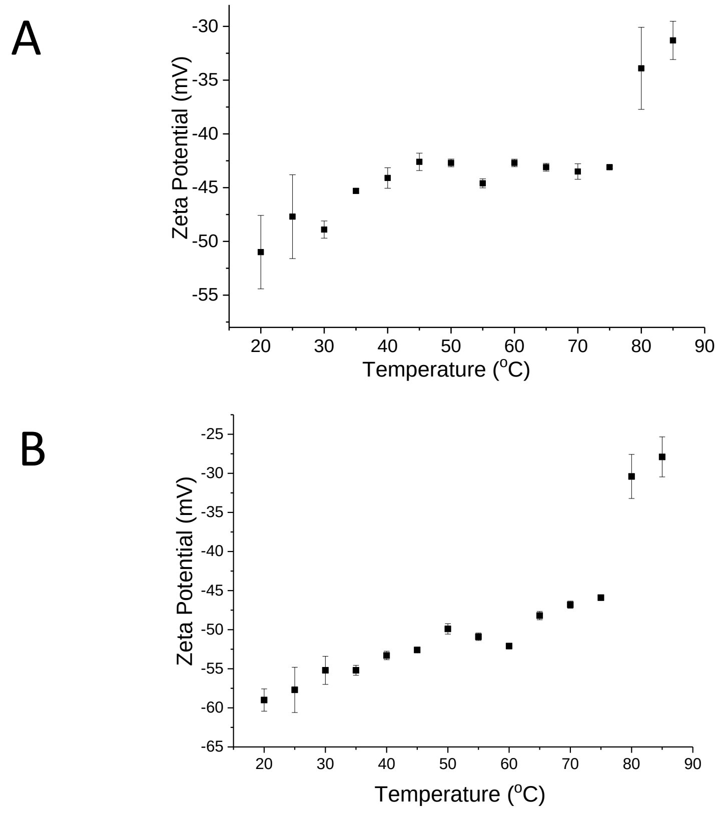

Figure S8. Zeta Potential values for (A) 398 nm polymersomes and (B) 53 nm polymersomes monitored from 25 to 90 °C.

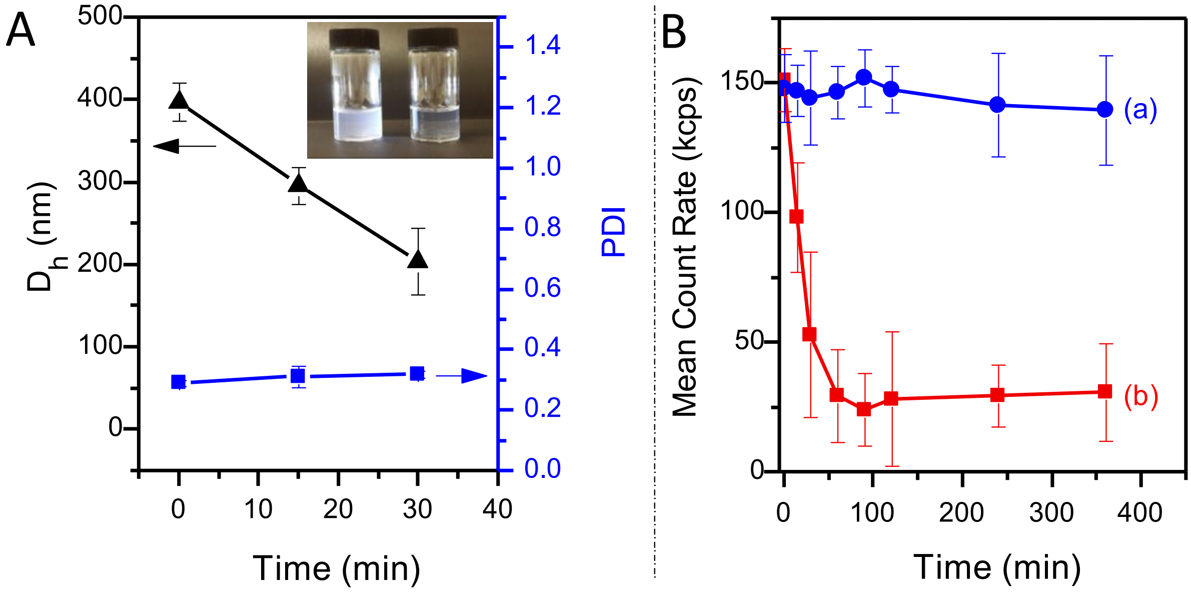

Figure S9. (A) Lipase catalysed biodegradation of polyester vesicles (Table 1, #4) monitored by DLS. Conditions: Pseudomonas cepacia lipase at 37 °C, the concentration of vesicles and lipase were 0.21 mg/mL and 100 unit/mL in deionized water, respectively. Inset: photos of the polyester vesicle solution before (left) and after (right) degradation; (B) Mean count rate variation of the polyester vesicle solutions during the experimental period with (b) or without (a) the presence of lipase.

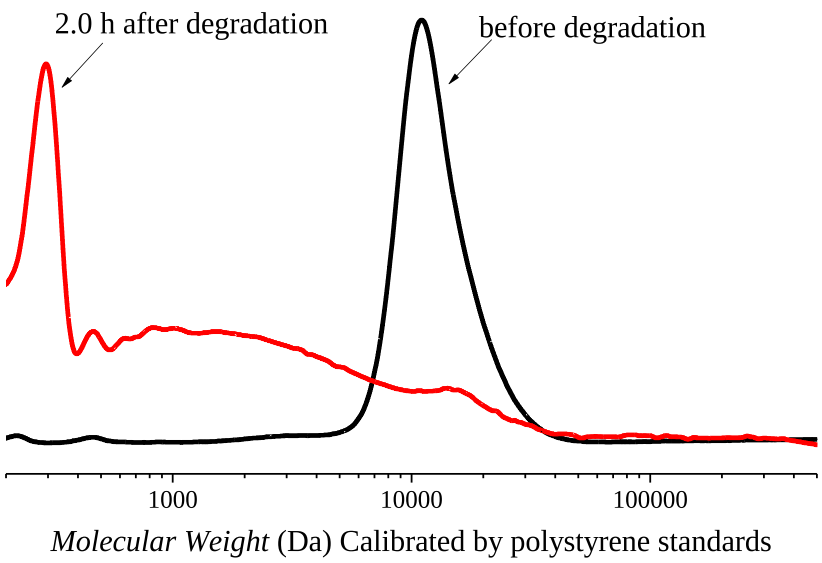

Figure S10. SEC traces the block copolyesters before and after lipase degradation.

Table S4. M_n and $Đ$ of the block copolyesters during lipase degradation experiments.[a]

#	t (h)	M_n (kg mol^{-1}), ($Đ$)[b]
1	0.0	16.5, (1.18)
2	2.0	1.4, (3.14)
3	4.0	–

[a)] Reaction conditions: DI Water, [PCL_{54}-*b*-PE_7] = 0.23 mg mL^{-1}; [Lipase] = 7.7 unit mL^{-1}; [b)] determined by SEC in THF at 30 °C, calibrated using narrow molar mass polystyrene standards.

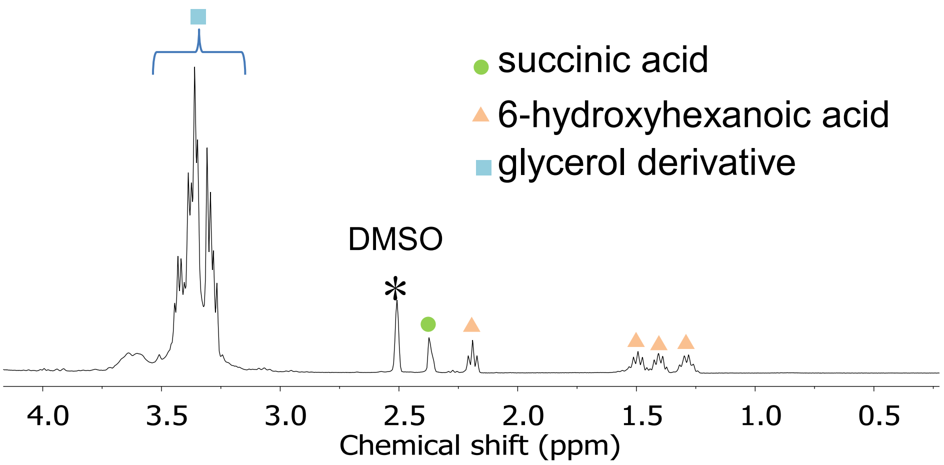

Figure S11. ^{1}H NMR spectrum of the degradation products of PCL_{54}-*b*-PE_7 (Table 1, #5) in DMSO-d_6.

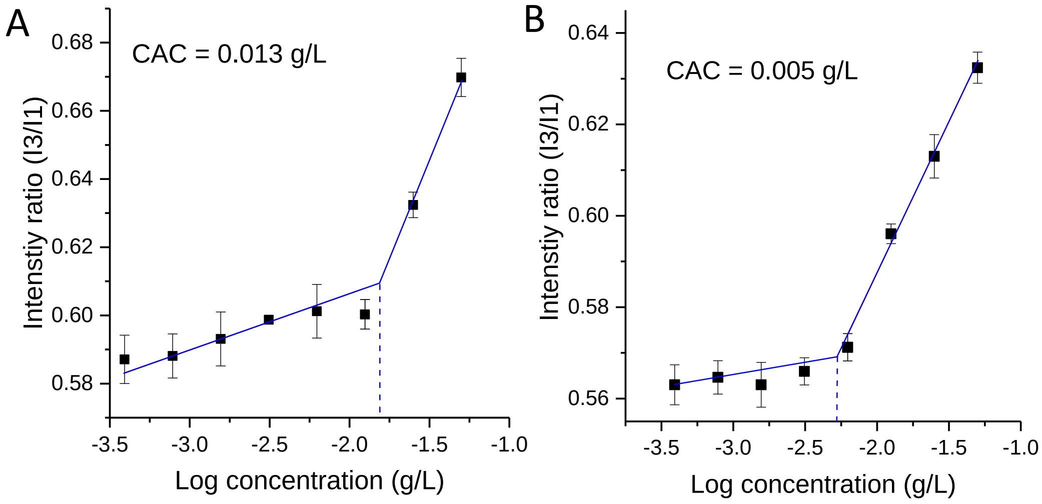

Figure S12. Variations of pyrene I3/I1 intensity ratio as a function of logarithm of polymer concentration (g/L) for (A) PCL_{38}-*b*-PE_7 and (B) PCL_{54}-*b*-PE_7 in DI water.

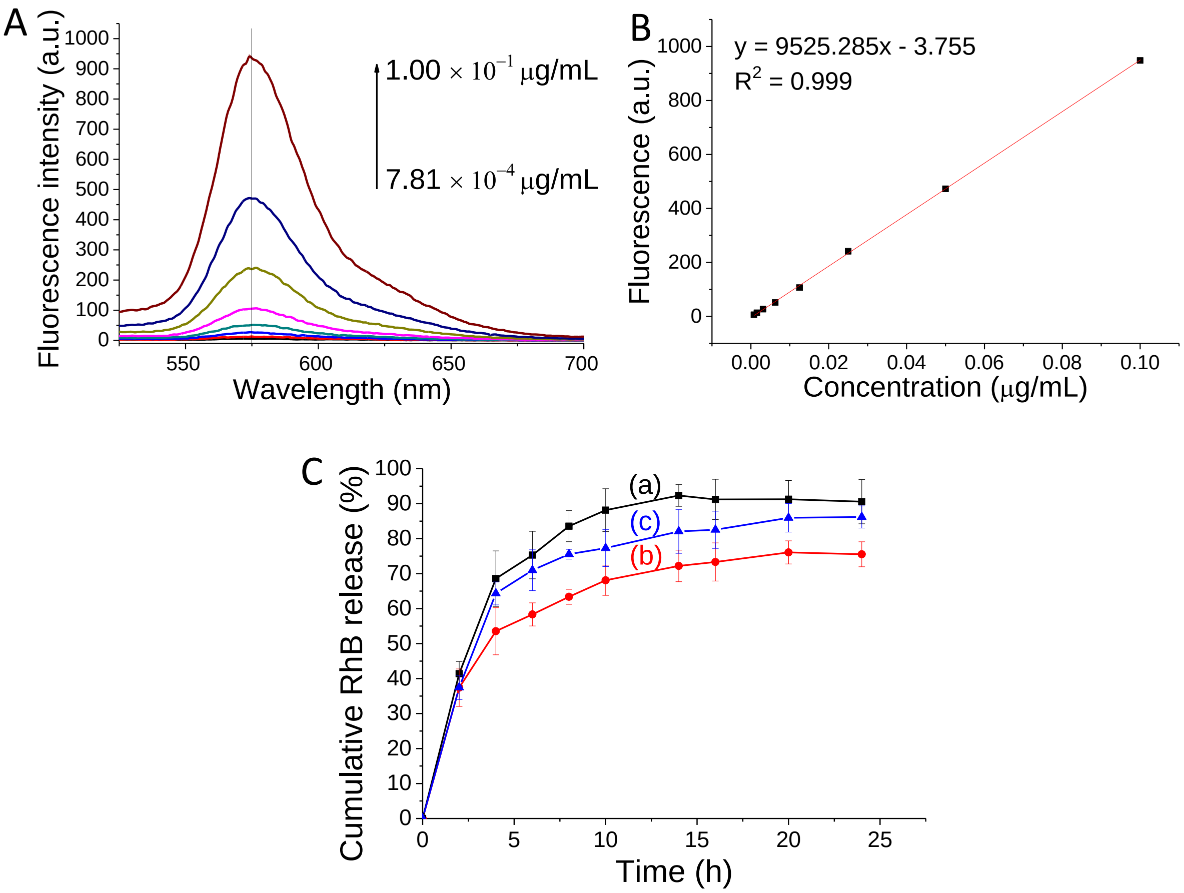

Figure S13. The fluorescence spectrum (A) and calibration (B) for Rhodamine B (RhB) in PBS solution (10.00 mM, pH 7.4), λ_{ex} = 510 nm; (C) Cumulative drug release profiles of RhB-loaded polymersomes. Reaction conditions: 37 °C, in PBS buffer (10.0 mM, pH 7.4): (a) free RhB; (b) RhB loaded polymersomes; (c) RhB loaded polymersomes with the presence of lipase.

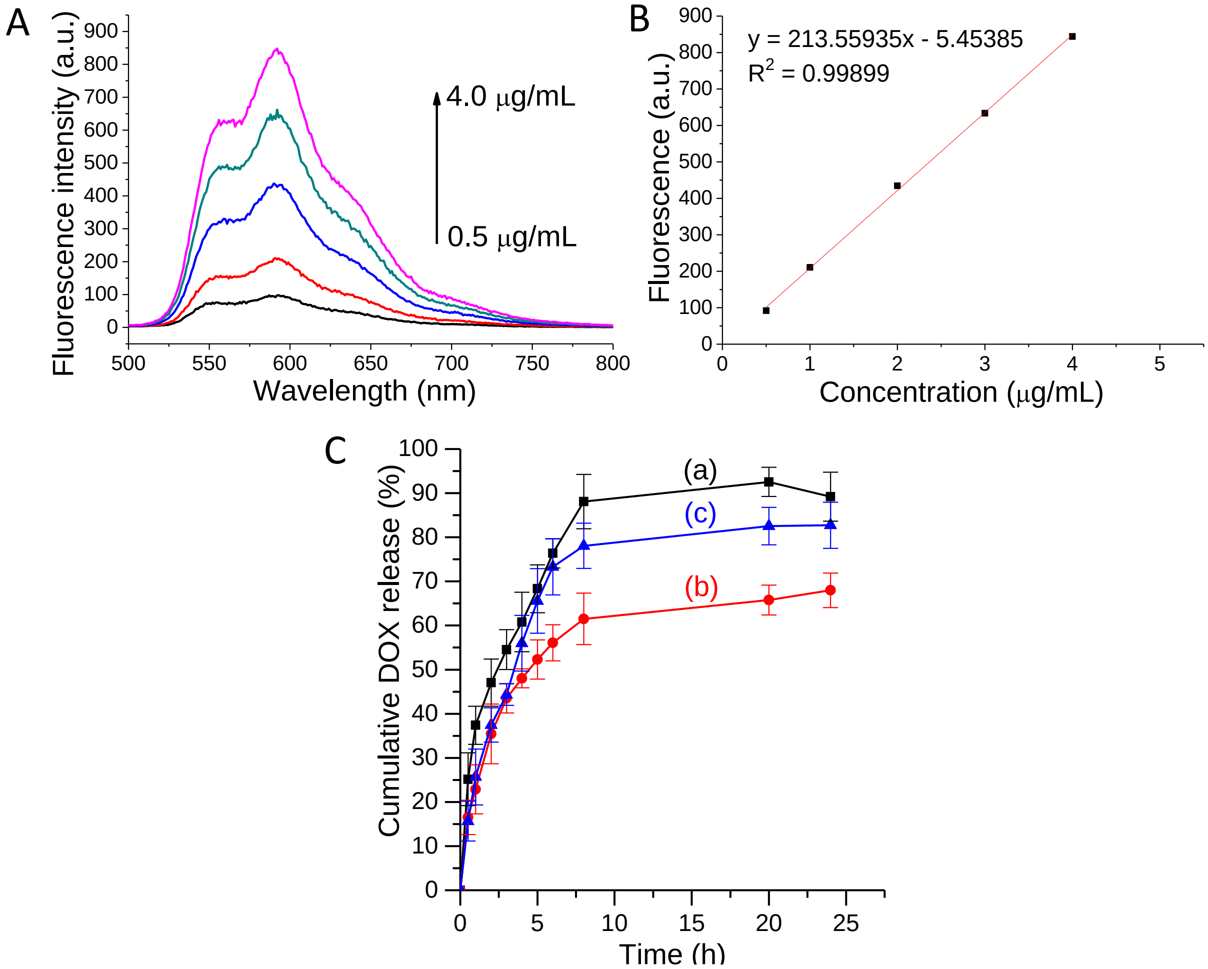

Figure S14. The fluorescence spectrum (A) and calibration (B) for DOX•HCl in PBS solution (10.0 mM, pH 7.4). (λ_{ex} = 461 nm); (C) Cumulative drug release profiles of DOX-loaded polymersomes. Reaction conditions: 37 °C, in PBS buffer (10.0 mM, pH 7.4): (a) free DOX; (b) DOX loaded polymersomes; (c) DOX loaded polymersomes with the presence of lipase.

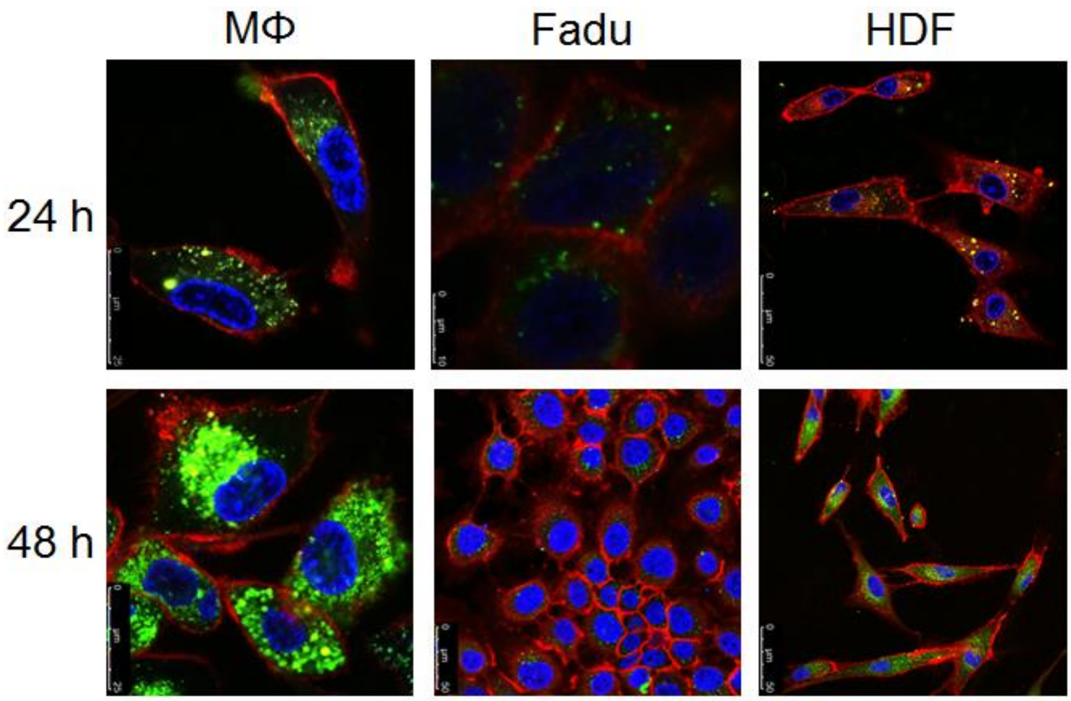

Figure S15. Confocal images of FaDu, MΦ and HDF incubated with polymersomes (53 nm) for 24 and 48 h. The quantification of polymersome uptake is shown in Figure 3B. The cell nuclei were stained with DAPI in blue and far-red CellMask™ was used for cell membrane staining.

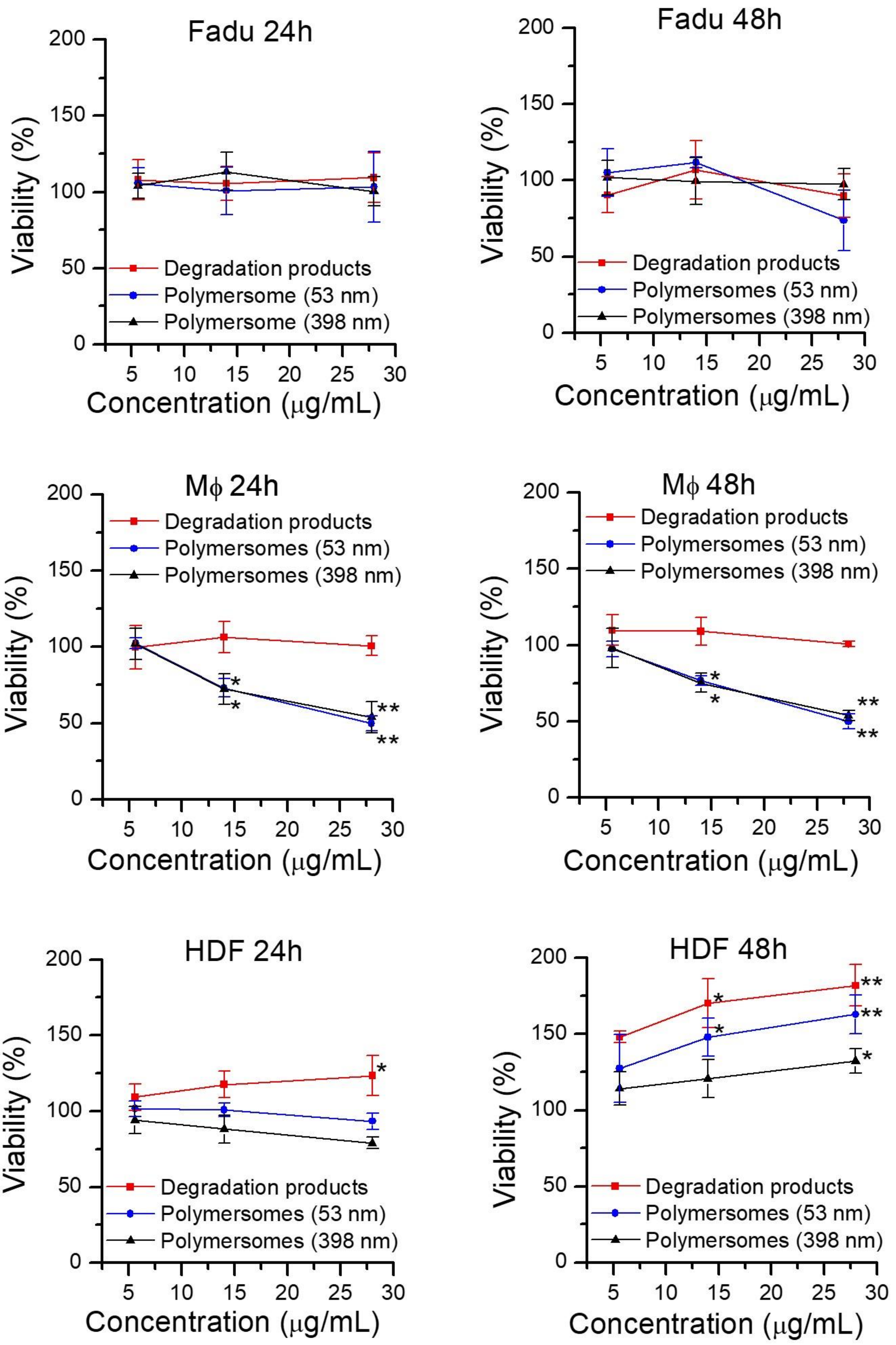

Figure S16. Viability (MTT) assay of cells incubated at increasing concentrations of degradation products (red), polymersomes (53 nm) (blue), and polymersomes (398 nm) (black) for 24 and 48 hours. All experiments were carried out as three independent replicates, followed by *t*-test statistical analyses. *$p<0.05$ and **$p<0.01$.

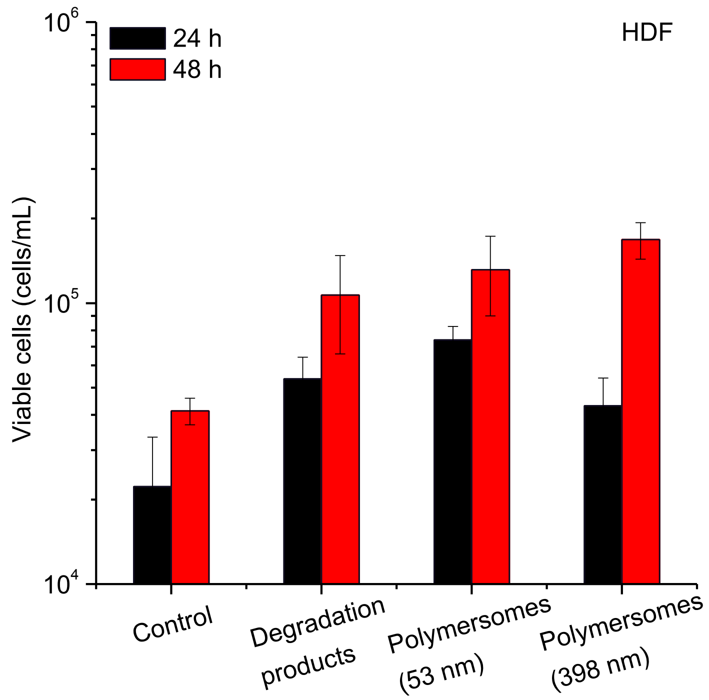

Figure S17. Proliferation assays of HDF. Degradation products, polymersomes (53 nm) and polymersomes (398 nm) induced hyperproliferation.

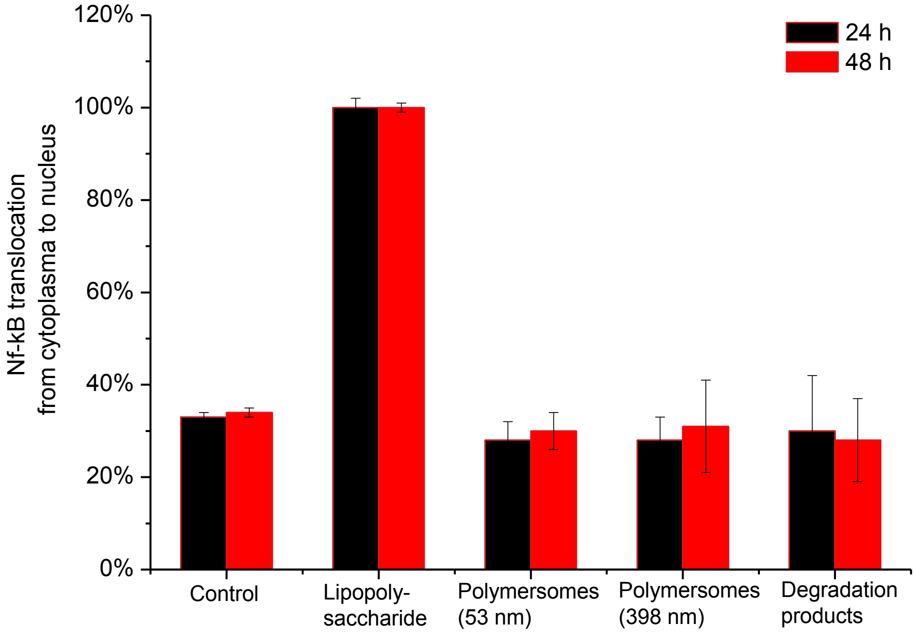

Figure S18. Nf-kB translocation quantification of MΦ treated with lipopolysaccharide (as the Nf-kB activator), polymersomes (53 nm), polymersomes (398 nm) and degradation products over 48 h.

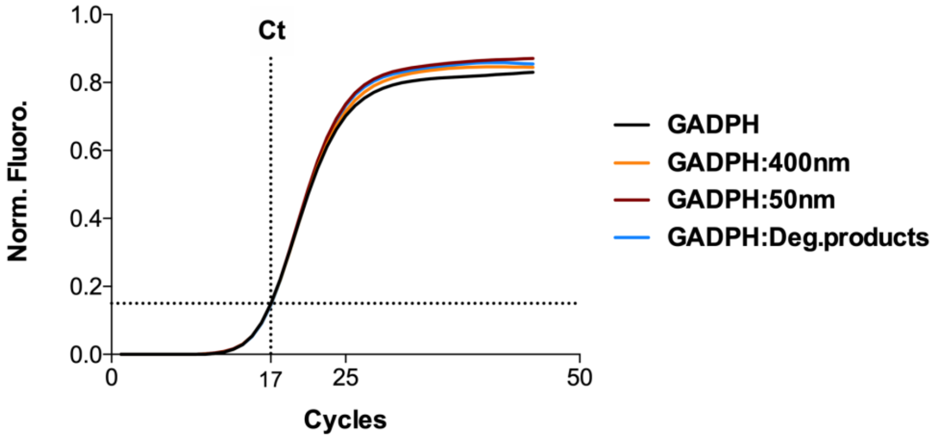

Figure S19. qPCR expression profiles of GADPH in all experimental groups.

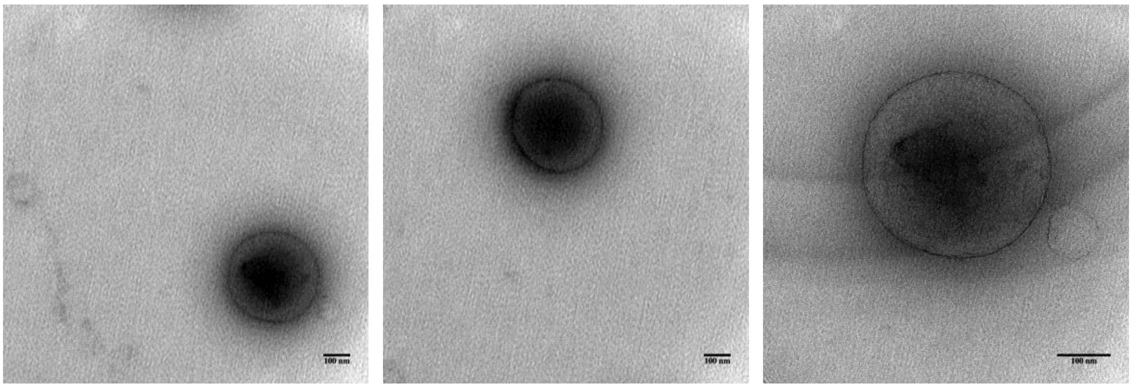

Figure S20. TEM representative images of a single polymersome made of PCL_{54}-*b*-PE_7 (scale bar 100 nm).

References:

[1] Chen, W. Q.; Du, J. Z. *Sci. Rep.* **2013,** 3, 2162.

[2] Daigneault, M.; Preston, J. A.; Marriott, H. M.; Whyte, M. K. B.; Dockrell, D. H. *PLoS One* **2010,** 5, e8668.

[3] Park, E. K.; Jung, H. S.; Yang, H. I.; Yoo, M. C.; Kim, C.; Kim, K. S. *Inflammation Res.* **2007,** 56, 45-50.

[4] Bornscheuer, U.; Reif, O.-W.; Lausch, R.; Freitag, R.; Scheper, T.; Kolisis, F. N.; Menge, U. *Biochim. Biophys. Acta, Gen. Subj.* **1994,** 1201, 55-60.

[5] a) Hatzakis, E.; Archavlis, E.; Dais, P. *J. Am. Oil. Chem. Soc.* **2007,** 84, 615-619; b) Spyros, A.; Argyropoulos, D. S.; Marchessault, R. H. *Macromolecules* **1997,** 30, 327-329.

[6] a) Lutz, J.-F.; Weichenhan, K.; Akdemir, Ö.; Hoth, A. *Macromolecules* **2007,** 40, 2503-2508; b) Fan, L.; Lu, H.; Zou, K.; Chen, J.; Du, J. *Chem. Commun.* **2013,** 49, 11521-3.

[7] O'Brien, R. W.; Midmore, B. R.; Lamb, A.; Hunter, R. J. *Faraday Discuss. Chem. Soc.* **1990,** 90, 301-312.
